# Rosacea-like facial rash related to metformin administration in a young woman

**DOI:** 10.1186/2050-6511-15-3

**Published:** 2014-02-08

**Authors:** Laura Mumoli, Antonio Gambardella, Angelo Labate, Elena Succurro, Giovambattista De Sarro, Franco Arturi, Luca Gallelli

**Affiliations:** 1 Department of Medical and Surgical Science, Division of Neurology, University “Magna Graecia”- Mater Domini Hospital of Catanzaro, Catanzaro, Italy; 2 Department of Medical and Surgical Science, Division of Internal Medicine, University “Magna Graecia”- Mater Domini Hospital of Catanzaro, Catanzaro, Italy; 3 Department of Health Science, Clinical Pharmacology and Pharmacovigilance Unit and Pharmacovigilance’s Centre Calabria Region, University “Magna Graecia”- Mater Domini Hospital of Catanzaro, Catanzaro, Italy; 4 Chair of Internal Medicine, Department of Medical and Surgical Science, School of Medicine, University of Catanzaro, “Mater Domini” University Hospital, Viale Europa – Germaneto, 88100, Catanzaro, Italy

**Keywords:** Facial skin rash, Metformin, Adverse drug reaction, Differential diagnosis

## Abstract

**Background:**

Since the skin represents a common site of adverse drug reactions, few data are reported at this time regarding the development of skin rash during the treatment with antidiabetic drugs.

**Case presentation:**

We report a 29-year old woman that developed a facial skin rash during the treatment with metformin. Clinical and laboratory findings excluded the presence of systemic diseases, but several diagnosis and many drugs were administered without clinical improvement. The self-dismission of metformin induced an improvement of symptoms, while the re-challenge documented an impairments of skin rash. The Naranjo probability scale suggested a probable association between metformin and skin rash and metformin was definitively dismissed.

**Conclusion:**

We report for the first time a non vasculitis facial skin manifestation related to metformin in a young woman. However, this case may emphasizes the need to consider the ADRs as a differential diagnosis in order to reduce medical errors and the related medical costs.

## Background

Several drugs are able to induce the development of adverse drug reactions (ADRs), and usually the skin represents a common site of manifestation [1-5]. However, few data are reported at this time regarding the development of skin rash during the treatment with antidiabetic drugs [6-9]. Salem and coworkers [7], described a leukocytosis vasculitis with purpuric necrotizing eruption in lower legs in a young woman during metformin’s treatment.

In this paper we describe for the first time a young woman that developed a rosacea-like facial skin rash during the treatment with metformin.

## Case presentation

**Figure 1:**
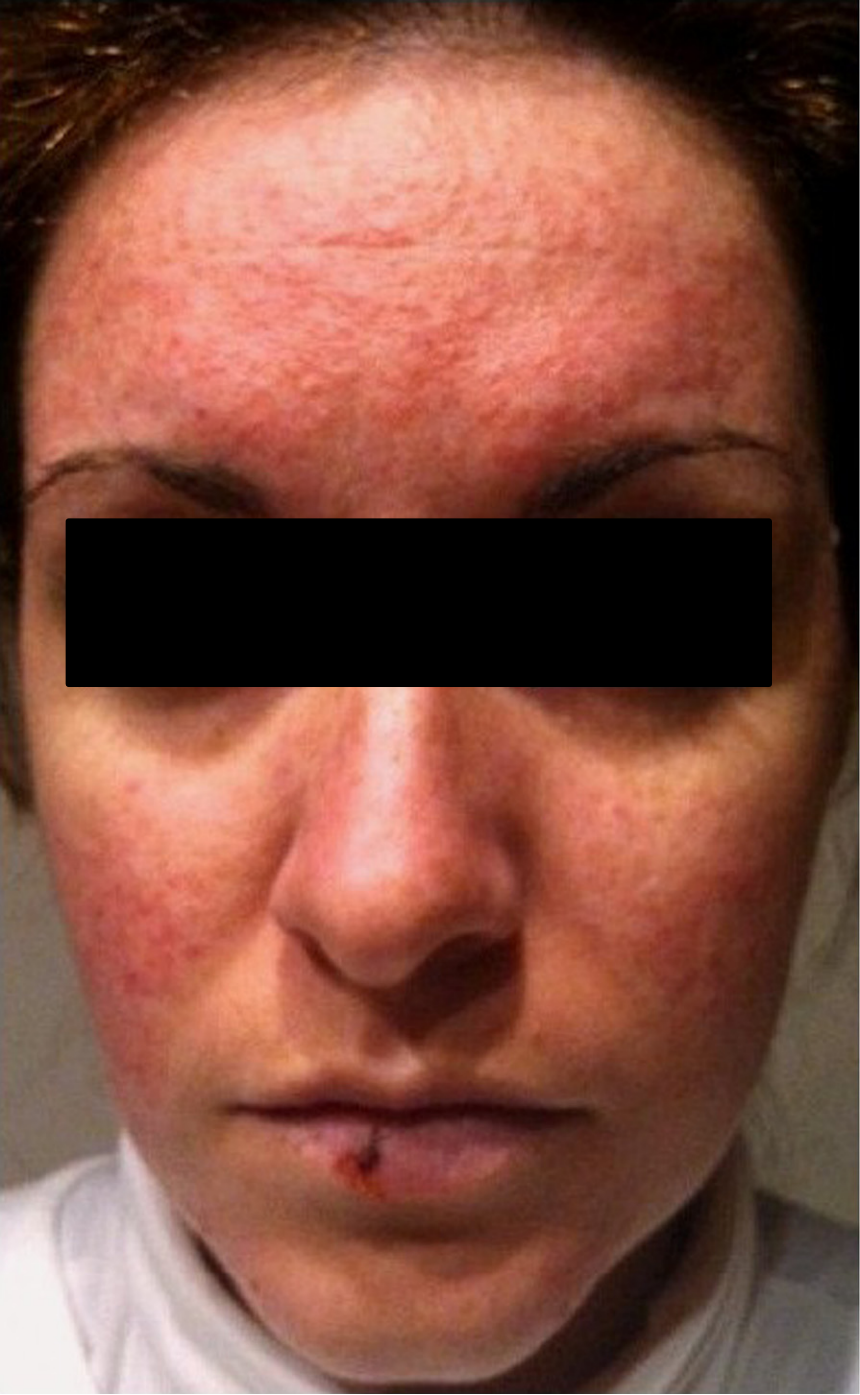
Skin rash during metformin treatment.

On December 2012, a 29-year-old woman presented to our observation for facial cutaneous rash that had appeared about 10 months earlier. She had only a past history of allergy to penicillin. Medical history was unremarkable until February 2012, when was made her a diagnosis of impaired glucose tolerance (IGT), insulin-resistance (evaluated by hyperinsulinemic euglycemic clamp) and subclinical hypothyroidism. For this reasons after we obtained the written informed consent, she started metformin (500 mg/12 h), used off-label, plus levothyroxine (50 μg/die). Two days after the beginning of this treatment she noticed intense pruritus and burning in the center of the face. In about 1 month her skin rash worsened in severity and the eruption involved the whole face (except for orbicularis oculi), in particular malar areas and forehead like a butterfly with papules and teleangectasies (see Figure 1). During this time she was not taking any pharmacological or herbal products except for metformin and levothyroxine. Firstly a dermatologist diagnosed a rosacea and prescribed both minocycline and metronidazole for 1 month, without any benefit. The persistence of symptoms induced a new clinical examination and another dermatologist hypothesized a probable subacute cutaneous lupus like-syndrome and treated with cetirizine, vitamin E, total-block sunscreens and lincomicine, without clinical effects. A new dermatologist diagnosed a probable toxic mixoedema thyroid-based disease, so deflazacort (30 mg/day for 1 month) was started with a transient moderate improvement of symptoms that reappeared when the therapy was finished.

On December 02^nd^ 2012, the patient forgot to take the metformin treatment and she noted a moderate improvement of pruritus, and due to this empiric experience she went to our observation. On admission clinical examination revealed the presence of erythema with papular eruption involving cheeks, glabella, perioral zone, until scalp and mandibular area. There was no involvement of neck, ears, shoulders, groin, thighs or knees. She was overweight (Body Mass Index = 28 kg/m^2^) and cardiopulmonary, abdominal, ophthalmologic systems were unremarkable. Laboratory findings (i.e. blood cells count, immunoglobulins, C3, C4, C-reactive protein, glucose, insulin, serum protein electrophoresis and urinalysis) were in normal range. Both an extensive autoimmune tests (i.e. antinuclear antineutrophil cytoplasmic, anti-Ro/SSA antibodies, anti double stranded-DNA antibodies cryoglobulins, rheumatoid factor) and infective serological screening (i.e. hepatitis B, C, helicobacter pylori) were negative too. A nailfold capillaroscopy showed a pattern of regular disposition of the capillary loops along with the nailbed.

**Figure 2:**
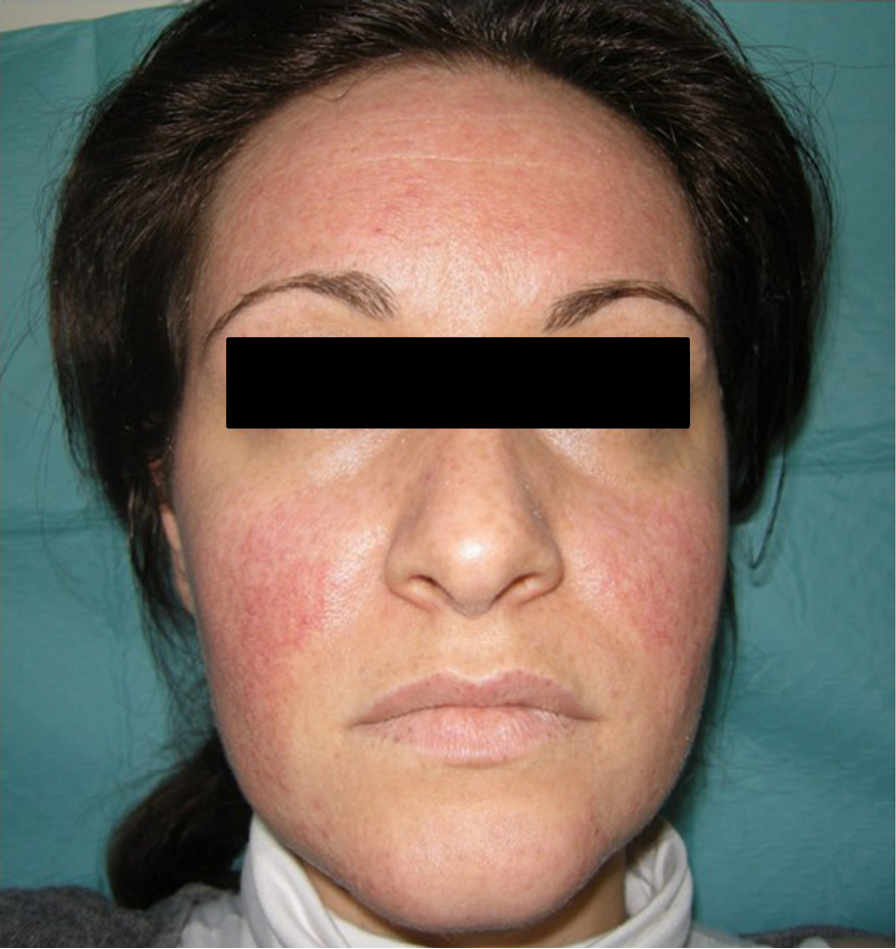
**Clinical evaluation 7 days after the dismission of metformin.** It is possible to see a decreased manifestation of facial skin rash, considering both papular rash and hyperemia.

**Figure 3:**
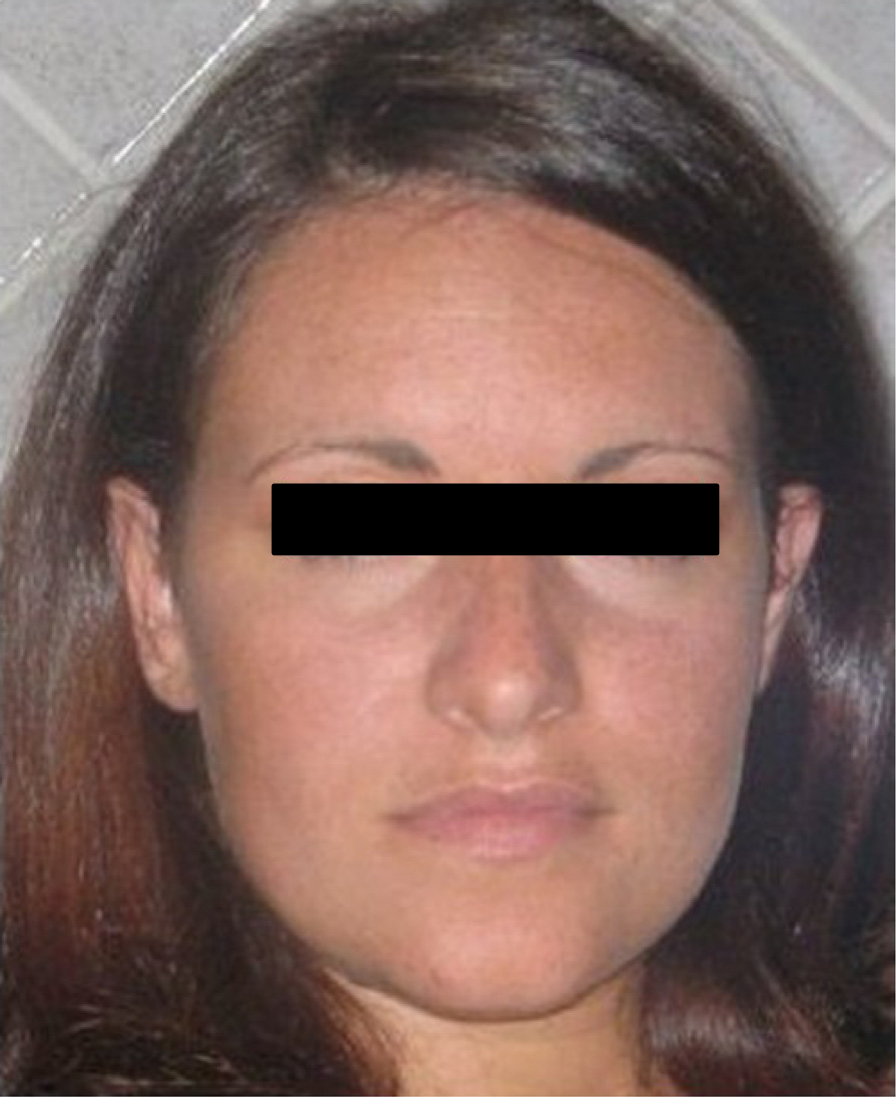
**Clinical evaluation performed 1 month after the dismission of metformin.** It is possible to evaluate a complete remission of facial skin rash.

In order to evaluate the association between metformin and facial rash, we re-administered metformin with an impairment of skin manifestation. According to the Naranjo probability scale [10], we documented a probable association (Naranjo Score 7/13) between facial rush and metformin administration. Metformin was stopped with a progressive remission of exanthema in about 1 month (Figures 2 and 3).

Type 2 diabetes mellitus is a serious and costly disease and the Diabetes Prevention Program Research Group demonstrated that treatment with metformin is able to reduce the incidence of diabetes in subjects with such risk factors including impaired glucose tolerance [11] and/or to reduce the insulin-resistance [12]. In agreement with these studies, after we advise the patient regarding the treatment and obtained the written informed consent, an off-label treatment with metformin was started. Metformin has been used clinically for many years with good safety profile, and rarely induces skin ADRs [6]. However, Salem and coworkers [7], described a leukocytosis vasculitis with purpuric necrotizing eruption in lower legs in a young woman during metformin’s treatment.

## Conclusion

In the present case, we report for the first time a non vasculitis facial skin eruption during the treatment with metformin. Using the Naranjo probability scale, we documented a probable association between metformin and the skin manifestation. We are not able to explain the pathogenesis of this manifestation, since capillaroscopy excluded a vasculitis pathogenesis, because the patient denied consent to biopsy. However, probably an allergic pathogenesis may be suggested.

Nevertheless the aim of the present case is to describe a rare case of ADR related to metformin treatment in order to increase the safety information concerning this drug that, in addition to the treatment of Type 2 diabetes mellitus, it is usually used, off-label, for both impaired glucose tolerance (IGT) and insulin-resistance treatment and for the polycystic ovary syndrome (PCOS) management. Indeed we would remark that the diagnosis of ADR is not easy and often unreported and misdiagnosed [13,14]. Finally, this case may emphasizes the need to consider the ADRs as a differential diagnosis in order to reduce medical errors and the related medical costs.

## Consent

Written informed consent was obtained from the patient for publication of this Case report and any accompanying images. A copy of the written consent is available for review by the Editor of this journal”.

## Abbreviations

ADRs: Adverse drug reactions; IGT: Impaired glucose tolerance; PCOS: Polycystic ovary syndrome.

## Competing interests

The authors declare that they have no competing interests.

## Authors’ contributions

LM, LG, ES, and FA were the treating physicians of the patient reported. LM, LA, ES and FA evaluated the test results and designed the manuscript and figures. LM, LA, AG, ES, GDS, LG and FA have participated in the discussion and in writing of the submitted manuscript. All authors read and approved the final version of manuscript.

## Pre-publication history

The pre-publication history for this paper can be accessed here:

http://www.biomedcentral.com/2050-6511/15/3/prepub
